# Supplementary material for: Structure-Based Identification of Natural Products as SARS-CoV-2 Mpro Antagonist from Echinacea angustifolia Using Computational Approaches
Source: Viruses. 2021 Feb 15;13(2):305. doi: 10.3390/v13020305 (PMC7919488; doi:10.3390/v13020305)
Supplement: Supplementary file 1 [file viruses-13-00305-s001.pdf]

Supplementary Materials

Table S1. List of natural products from *Echinacea angustifolia* docked by extra precision method at the active pocket of SARS-CoV-2 M^{pro}.

S.no.	Natural products	CID	RMSD (Å)	Docking score (kcal/mol)	Glide energy (kcal/mol)
1	Echinacoside	5281771	0.008	-14.11	-84.854
2	Quercetagetin 7-Glucoside	44259796	0.008	-13.79	-60.593
3	Levan N	440946	0.05	-12.12	-61.748
4	Inulin from chicory	16219508	0.03	-11.72	-46.248
5	1,3-Dicaffeoylquinic Acid	6537500	0.011	-9.68	-58.238
6	Cynaroside	5280637	0.013	-8.90	-55.188
7	Sucrose	5988	0.012	-8.84	-35.726
8	Chlorogenic acid	1794427	0.04	-8.81	-45.066
9	Isochlorogenic acid	5315832	0.04	-8.69	-45.917
10	Astragalin	5282102	0.006	-8.56	-53.309
11	Cynarin	5281769	0.006	-8.43	-55.218
12	Isorhamnetin	5281654	0.029	-7.87	-40.679
13	Chicoric-Acid	5134221	0.046	-7.61	-46.919
14	Echinacin	6439941	0.044	-7.17	-60.869
15	Quercetin	5280343	0.011	-6.70	-40.152
16	Kaempferol	5280863	0.013	-6.86	-37.796
17	Luteolin	5280445	0.007	-6.71	-38.336
18	Echinamine B	11587035	0.01	-6.48	-32.479
19	Caftaric acid	6440397	0.017	-5.87	-34.281
20	Ethinyl estradiol	5991	0.001	-5.18	-32.908
21	Tussilagine	185071	0.017	-4.83	-30.357
22	Sitosterol-3-O-Beta-D-Glucoside	5742590	0.011	-4.21	-33.87
23	Apigenin	5280443	0.032	-4.18	-34.421
24	Isotussilagine	149801	0	-4.02	-29.252
25	Beta-Sitosterol	222284	0.007	-3.93	-32.512
26	Echinacein	131751109	0.028	-3.82	-32.294
27	Germacrene-D	91723653	0.021	-3.59	-21.114
28	Echinolone	6441887	0.025	-3.56	-28.23
29	Bornyl-Acetate	93009	0.028	-3.56	-24.979
30	Linolenic Acid	5280934	0.043	-3.38	-33.741
31	Behenic-Acid-Ethyl-Ester	22199	0.019	-3.25	-31.621
32	Linoleic-Acid	3931	0.032	-3.24	-30.778
33	Caryophyllene-Epoxide	14350	0	-3.09	-22.858
34	Pentadec-8-En-2-One	12811248	0.043	-3.09	-25.838
35	Caryophyllene	5281515	0.009	-3.07	-22.896
36	Dodeca-2,4-dien-1-yl-isovalerate	22041880	0.042	-2.67	-30.509
37	Palmitic acid	985	0.02	-2.51	-28.545
38	N, N-Dimethylisobutylamine	81653	0.008	-2.43	-13.834
39	N-Isobutyl-(2E,4E)-dodecadienamide	6443006	0.035	-2.29	-29.024
40	Doceca-2e,4e,8z,10e-z-tetraenoic-acid-isobutylamides	11413953	0.005	-2.17	-29.214
41	Trideca-1-11-diene-3-5-7-9-tetrayne	5322026	0.047	-2.12	-19.177
42	oleic acid	445639	0.045	-2.09	-28.055
43	Ethylene oxide	6354	0	-1.92	-8.399
44	*N*-(2-methylpropyl) undeca-2,4-dien-8,10-diynamide	593849	0.018	-1.83	-29.889
45	Trideca-1,3-diene-5,7,9,11-tetrayn	528755	0.045	-1.82	-16.299
46	Tridec-1-ene-3-5-7-9-11-pentayne	441552	0.042	-1.80	-16.112
47	1-pentadecene	25913	0.02	-1.39	-27.061
48	Betaine	247	0.003	-1.32	-10.184
49	Myristic-acid	11005	0.035	-0.90	-22.899
50	Glycine-betaine	247	0.007	0.90	-11.928

Table S2: Optimized geometry parameters for the natural product Echinacoside.

S.no.	Atom	Bond Atom	Bond Length (Å)	Angle Atom	Angle (°)	2nd Angle Atom	2nd Angle (°)	2nd Angle Type
1	C(43)	-	-	-	-	-	-	-
2	C(45)	C(43)	1.402	-	-	-	-	-
3	C(46)	C(43)	1.396	C(45)	118.254	-	-	-

4	C(48)	C(45)	1.387	C(43)	120.733	C(46)	0.087	Dihedral
5	C(49)	C(46)	1.395	C(43)	121.19	C(45)	-0.057	Dihedral
6	C(50)	C(48)	1.403	C(45)	120.568	C(43)	-0.105	Dihedral
7	O(17)	C(48)	1.377	C(45)	124.502	C(50)	114.929	Pro-S
8	O(18)	C(50)	1.361	C(48)	120.622	C(49)	120.396	Pro-S
9	C(41)	C(43)	1.512	C(45)	120.451	C(46)	121.269	Pro-S
10	C(39)	C(41)	1.533	C(43)	112.276	C(45)	-85.561	Dihedral
11	O(6)	C(39)	1.444	C(41)	109.949	C(43)	174.345	Dihedral
12	C(25)	O(6)	1.398	C(39)	117.405	C(41)	120.851	Dihedral
13	O(2)	C(25)	1.413	O(6)	110.026	C(39)	51.869	Dihedral
14	C(24)	C(25)	1.538	O(2)	110.552	O(6)	111.928	Pro-S
15	C(21)	C(24)	1.531	C(25)	110.453	O(2)	57.453	Dihedral
16	C(22)	C(21)	1.529	C(24)	108.854	C(25)	-53.459	Dihedral
17	C(23)	O(2)	1.427	C(25)	112.6	O(6)	174.391	Dihedral
18	O(1)	C(21)	1.428	C(22)	108.35	C(24)	111.481	Pro-S
19	C(26)	O(1)	1.43	C(21)	115.068	C(22)	150.93	Dihedral
20	O(3)	C(26)	1.4	O(1)	112.759	C(21)	-66.845	Dihedral
21	C(27)	C(26)	1.538	O(1)	105.596	O(3)	113.701	Pro-R
22	C(29)	C(27)	1.526	C(26)	110.977	O(1)	76.881	Dihedral
23	C(30)	C(29)	1.53	C(27)	110.589	C(26)	50.049	Dihedral
24	C(31)	O(3)	1.447	C(26)	116.435	O(1)	-67.575	Dihedral
25	O(4)	C(22)	1.431	C(21)	110.582	C(23)	106.825	Pro-S
26	O(7)	C(24)	1.423	C(21)	109.686	C(25)	109.579	Pro-R
27	O(9)	C(27)	1.431	C(26)	111.179	C(29)	105.45	Pro-S
28	O(10)	C(29)	1.426	C(27)	111.491	C(30)	110.934	Pro-S
29	O(11)	C(30)	1.419	C(29)	110.085	C(31)	108.636	Pro-R
30	C(28)	C(23)	1.517	O(2)	108.661	C(22)	113.495	Pro-S
31	C(37)	C(31)	1.516	O(3)	107.011	C(30)	113.34	Pro-R
32	C(40)	O(4)	1.386	C(22)	120.029	C(21)	126.945	Dihedral
33	C(42)	C(40)	1.474	O(4)	116.041	C(22)	-45.101	Dihedral
34	O(16)	C(40)	1.204	O(4)	118.19	C(42)	125.694	Pro-R
35	C(44)	C(42)	1.347	C(40)	119.864	O(4)	169.695	Dihedral
36	C(47)	C(44)	1.458	C(42)	127.397	C(40)	176.741	Dihedral
37	C(51)	C(47)	1.406	C(44)	118.378	C(42)	164.054	Dihedral
38	C(52)	C(47)	1.404	C(44)	122.854	C(51)	118.763	Pro-S
39	C(53)	C(51)	1.386	C(47)	121.064	C(44)	-179.957	Dihedral
40	C(54)	C(52)	1.389	C(47)	120.442	C(44)	-179.616	Dihedral
41	C(55)	C(53)	1.404	C(51)	119.263	C(47)	-0.631	Dihedral
42	O(19)	C(53)	1.36	C(51)	120.17	C(55)	120.566	Pro-R
43	O(20)	C(55)	1.372	C(53)	115.133	C(54)	124.51	Pro-R
44	O(5)	C(28)	1.43	C(23)	109.733	O(2)	-69.684	Dihedral
45	C(32)	O(5)	1.394	C(28)	113.99	C(23)	-175.632	Dihedral
46	O(8)	C(32)	1.425	O(5)	108.288	C(28)	-66.843	Dihedral
47	C(33)	C(32)	1.533	O(5)	107.983	O(8)	111.218	Pro-R
48	C(34)	C(33)	1.522	C(32)	109.459	O(5)	174.399	Dihedral
49	C(35)	C(34)	1.53	C(33)	111.62	C(32)	-50.068	Dihedral
50	C(36)	O(8)	1.426	C(32)	112.979	O(5)	178.457	Dihedral
51	O(12)	C(33)	1.416	C(32)	110.444	C(34)	108.382	Pro-S
52	O(13)	C(34)	1.417	C(33)	109.173	C(35)	110.035	Pro-R
53	O(14)	C(35)	1.422	C(34)	105.695	C(36)	112.671	Pro-S
54	C(38)	C(36)	1.525	O(8)	104.874	C(35)	113.385	Pro-S
55	O(15)	C(38)	1.433	C(36)	108.306	O(8)	-176.983	Dihedral
56	H(92)	C(45)	1.087	C(43)	119.95	C(48)	119.315	Pro-S
57	H(93)	C(46)	1.085	C(43)	119.641	C(49)	119.168	Pro-R
58	H(94)	C(49)	1.083	C(46)	121.296	C(50)	118.43	Pro-R
59	H(95)	C(51)	1.083	C(47)	120.482	C(53)	118.437	Pro-S
60	H(96)	C(52)	1.082	C(47)	120.541	C(54)	118.994	Pro-S
61	H(98)	C(54)	1.086	C(52)	120.15	C(55)	119.735	Pro-S
62	H(89)	C(42)	1.083	C(40)	117.595	C(44)	122.519	Pro-S
63	H(91)	C(44)	1.086	C(42)	116.543	C(47)	116.05	Pro-S
64	H(56)	C(21)	1.096	O(1)	109.134	C(22)	109.158	Pro-R
65	H(57)	C(22)	1.092	O(4)	110.698	C(21)	109.641	Pro-R
66	H(58)	C(23)	1.101	O(2)	109.48	C(22)	107.827	Pro-R
67	H(59)	C(24)	1.099	O(7)	110.326	C(21)	107.966	Pro-S
68	H(60)	C(25)	1.1	O(2)	110.486	O(6)	104.706	Pro-R
69	H(61)	C(26)	1.091	O(1)	109.596	O(3)	104.923	Pro-S
70	H(62)	C(27)	1.095	O(9)	110.478	C(26)	108.599	Pro-R
71	H(65)	C(29)	1.092	O(10)	106.13	C(27)	109.685	Pro-R
72	H(66)	C(30)	1.1	O(11)	110.936	C(29)	108.9	Pro-S
73	H(67)	C(31)	1.095	O(3)	109.159	C(30)	108.589	Pro-S
74	H(68)	C(32)	1.104	O(5)	110.231	O(8)	109.24	Pro-S
75	H(69)	C(33)	1.098	O(12)	111.431	C(32)	107.819	Pro-R
76	H(70)	C(34)	1.101	O(13)	110.315	C(33)	107.928	Pro-S
77	H(71)	C(35)	1.101	O(14)	110.72	C(34)	107.894	Pro-R
78	H(72)	C(36)	1.1	O(8)	110.811	C(35)	108.733	Pro-R
79	H(63)	C(28)	1.092	O(5)	109.955	C(23)	108.768	Pro-R
80	H(64)	C(28)	1.096	O(5)	109.876	C(23)	108.987	Pro-S
81	H(78)	C(38)	1.097	O(15)	111.277	C(36)	108.326	Pro-R
82	H(79)	C(38)	1.096	O(15)	111.085	C(36)	109.152	Pro-S
83	H(80)	C(39)	1.093	O(6)	106.911	C(41)	110.29	Pro-S

84	H(81)	C(39)	1.091	O(6)	110.82	C(41)	111.438	Pro-R
85	H(87)	C(41)	1.095	C(39)	109.115	C(43)	109.737	Pro-S
86	H(88)	C(41)	1.095	C(39)	108.121	C(43)	110.445	Pro-R
87	H(73)	O(7)	0.966	C(24)	106.56	C(21)	161.86	Dihedral
88	H(74)	C(37)	1.091	C(31)	109.598	O(3)	177.765	Dihedral
89	H(75)	C(37)	1.092	C(31)	110.339	H(74)	109.009	Pro-S
90	H(76)	C(37)	1.093	C(31)	110.277	H(74)	108.934	Pro-R
91	H(77)	O(9)	0.962	C(27)	108.997	C(26)	69.202	Dihedral
92	H(82)	O(10)	0.967	C(29)	105.594	C(27)	-43.193	Dihedral
93	H(83)	O(11)	0.965	C(30)	106.028	C(29)	-45.285	Dihedral
94	H(84)	O(12)	0.964	C(33)	107.687	C(32)	60.137	Dihedral
95	H(85)	O(13)	0.964	C(34)	105.846	C(33)	175.86	Dihedral
96	H(86)	O(14)	0.967	C(35)	106.96	C(34)	-171.786	Dihedral
97	H(90)	O(15)	0.961	C(38)	108.74	C(36)	174.365	Dihedral
98	H(97)	O(17)	0.962	C(48)	110.084	C(45)	0.482	Dihedral
99	H(99)	O(18)	0.966	C(50)	107.759	C(48)	0.36	Dihedral
100	H(100)	O(19)	0.966	C(53)	107.999	C(51)	-179.641	Dihedral
101	H(101)	O(20)	0.962	C(55)	110.201	C(53)	179.758	Dihedral

Table S3: Optimized geometry parameters for the natural product Quercetagetin 7-Glucoside.

S.no.	Atom	Bond Atom	Bond Length (Å)	Angle Atom	Angle (°)	2nd Angle Atom	2nd Angle (°)	2nd Angle Type
1	C(23)	-	-	-	-	-	-	-
2	C(24)	C(23)	1.407	-	-	-	-	-
3	O(7)	C(23)	1.364	C(24)	121.864	-	-	-
4	C(21)	C(23)	1.391	O(7)	115.454	C(24)	122.675	Pro-S
5	C(25)	C(24)	1.414	C(23)	117.345	O(7)	179.085	Dihedral
6	C(26)	C(24)	1.48	C(23)	119.761	C(25)	122.891	Pro-S
7	C(20)	C(21)	1.388	C(23)	118.655	O(7)	-179.657	Dihedral
8	C(22)	C(25)	1.403	C(24)	120.538	C(23)	0.485	Dihedral
9	C(27)	O(7)	1.373	C(23)	120.858	C(21)	178.875	Dihedral
10	C(28)	C(26)	1.479	C(24)	113.809	C(23)	0.366	Dihedral
11	C(29)	C(27)	1.471	O(7)	112.118	C(28)	126.553	Pro-S
12	C(30)	C(29)	1.407	C(27)	119.658	O(7)	-34.108	Dihedral
13	C(31)	C(29)	1.403	C(27)	121.824	C(30)	118.509	Pro-S
14	C(32)	C(30)	1.383	C(29)	120.442	C(27)	179.856	Dihedral
15	C(33)	C(31)	1.393	C(29)	120.811	C(27)	179.997	Dihedral
16	C(34)	C(32)	1.407	C(30)	120.758	C(29)	-0.063	Dihedral
17	O(2)	C(20)	1.387	C(21)	119.965	C(22)	119.045	Pro-S
18	C(18)	O(2)	1.457	C(20)	117.201	C(21)	82.013	Dihedral
19	O(1)	C(18)	1.392	O(2)	108.86	C(20)	-142.066	Dihedral
20	C(16)	C(18)	1.537	O(1)	111.592	O(2)	109.027	Pro-S
21	C(14)	C(16)	1.544	C(18)	109.237	O(1)	-57.065	Dihedral
22	C(15)	C(14)	1.546	C(16)	110.301	C(18)	53.343	Dihedral
23	C(17)	O(1)	1.438	C(18)	115.627	O(2)	-61.315	Dihedral
24	O(8)	C(22)	1.372	C(20)	125.3	C(25)	114.841	Pro-R
25	O(9)	C(25)	1.345	C(22)	118.39	C(24)	121.07	Pro-R
26	O(10)	C(28)	1.361	C(26)	113.812	C(27)	123.864	Pro-S
27	O(12)	C(32)	1.374	C(30)	124.566	C(34)	114.675	Pro-S
28	O(13)	C(34)	1.357	C(32)	120.562	C(33)	120.397	Pro-R
29	O(3)	C(14)	1.434	C(15)	110.388	C(16)	112.113	Pro-S
30	O(4)	C(15)	1.441	C(14)	110.186	C(17)	108.184	Pro-S
31	O(5)	C(16)	1.414	C(14)	112.092	C(18)	110.482	Pro-R
32	C(19)	C(17)	1.532	O(1)	107.547	C(15)	114.593	Pro-R
33	O(11)	C(26)	1.217	C(24)	124.997	C(28)	121.191	Pro-R
34	O(6)	C(19)	1.409	C(17)	115.091	O(1)	56.76	Dihedral
35	H(46)	C(21)	1.081	C(20)	120.724	C(23)	120.603	Pro-R
36	H(49)	C(30)	1.084	C(29)	119.588	C(32)	119.97	Pro-R
37	H(50)	C(31)	1.083	C(29)	120.125	C(33)	119	Pro-S
38	H(52)	C(33)	1.083	C(31)	121.054	C(34)	118.506	Pro-S
39	H(35)	C(14)	1.093	O(3)	105.81	C(15)	109.486	Pro-R
40	H(36)	C(15)	1.096	O(4)	110.162	C(14)	108.77	Pro-R
41	H(37)	C(16)	1.091	O(5)	106.403	C(14)	109.235	Pro-S
42	H(38)	C(17)	1.098	O(1)	108.884	C(15)	107.232	Pro-S
43	H(39)	C(18)	1.09	O(1)	106.606	O(2)	109.604	Pro-R
44	H(40)	C(19)	1.102	O(6)	112.234	C(17)	107.226	Pro-R
45	H(41)	C(19)	1.092	O(6)	106.512	C(17)	107.862	Pro-S
46	H(42)	O(3)	0.968	C(14)	106.937	C(15)	-60.907	Dihedral
47	H(43)	O(4)	0.963	C(15)	108.954	C(14)	63.547	Dihedral
48	H(44)	O(5)	0.967	C(16)	107.419	C(14)	57.485	Dihedral
49	H(45)	O(6)	0.964	C(19)	107.01	C(17)	51.438	Dihedral
50	H(47)	O(8)	0.973	C(22)	113.296	C(20)	-12.48	Dihedral
51	H(48)	O(9)	0.969	C(25)	106.879	C(22)	-1.142	Dihedral
52	H(51)	O(10)	0.965	C(28)	109.102	C(26)	164.824	Dihedral
53	H(53)	O(12)	0.962	C(32)	110.297	C(30)	0.365	Dihedral
54	H(54)	O(13)	0.966	C(34)	107.967	C(32)	-0.1	Dihedral

Table S4: Optimized geometry parameters for the natural product Levan N.

S.no.	Atom	Bond Atom	Bond Length (Å)	Angle Atom	Angle (°)	2nd Angle Atom	2nd Angle (°)	2nd Angle Type
1	O(1)	-	-	-	-	-	-	-
2	C(17)	O(1)	1.418	-	-	-	-	-
3	C(18)	O(1)	1.446	C(17)	109.419	-	-	-
4	C(19)	C(17)	1.546	O(1)	103.444	C(18)	35.22	Dihedral
5	C(20)	C(18)	1.55	O(1)	106.613	C(17)	-19.907	Dihedral
6	O(2)	C(17)	1.408	O(1)	112.166	C(19)	107.567	Pro-R
7	O(6)	C(19)	1.408	C(17)	116.343	C(20)	110.374	Pro-R
8	O(7)	C(20)	1.418	C(18)	113.997	C(19)	115.082	Pro-R
9	C(24)	C(18)	1.524	O(1)	111.928	C(20)	111.77	Pro-S
10	C(31)	C(17)	1.534	O(1)	106.429	O(2)	112.717	Pro-R
11	O(3)	C(24)	1.431	C(18)	109.152	O(1)	45.81	Dihedral
12	C(21)	O(3)	1.41	C(24)	117.559	C(18)	144.711	Dihedral
13	O(4)	C(21)	1.426	O(3)	111.451	C(24)	-63.215	Dihedral
14	C(22)	C(21)	1.539	O(3)	105.923	O(4)	103.943	Pro-R
15	C(25)	C(22)	1.529	C(21)	100.834	O(3)	79.153	Dihedral
16	C(27)	O(4)	1.444	C(21)	110.802	O(3)	-90.318	Dihedral
17	O(8)	C(22)	1.406	C(21)	113.94	C(25)	114.516	Pro-R
18	O(9)	C(25)	1.418	C(22)	114.706	C(27)	115.518	Pro-S
19	C(32)	C(21)	1.528	O(3)	113.054	O(4)	105.939	Pro-S
20	C(33)	C(27)	1.514	O(4)	111.936	C(25)	114.124	Pro-S
21	O(13)	C(31)	1.418	C(17)	111.556	O(1)	-55.358	Dihedral
22	O(14)	C(32)	1.42	C(21)	113.16	O(3)	60.152	Dihedral
23	O(15)	C(33)	1.421	C(27)	108.424	O(4)	71.836	Dihedral
24	C(30)	O(2)	1.429	C(17)	117.479	O(1)	-57.58	Dihedral
25	C(23)	C(30)	1.519	O(2)	109.067	C(17)	169.744	Dihedral
26	O(5)	C(23)	1.442	C(30)	112.583	O(2)	-78.253	Dihedral
27	C(26)	C(23)	1.535	O(5)	105.034	C(30)	115.133	Pro-R
28	C(28)	C(26)	1.522	C(23)	101.557	O(5)	-30.849	Dihedral
29	C(29)	O(5)	1.464	C(23)	111.368	C(26)	9.496	Dihedral
30	O(10)	C(26)	1.42	C(23)	115.078	C(28)	114.566	Pro-R
31	O(11)	C(28)	1.404	C(26)	115.906	C(29)	112.273	Pro-S
32	O(12)	C(29)	1.386	O(5)	109.83	C(28)	111.336	Pro-R
33	C(34)	C(29)	1.535	O(5)	109.528	O(12)	109.855	Pro-R
34	O(16)	C(34)	1.417	C(29)	111.201	O(5)	79.743	Dihedral
35	H(35)	C(18)	1.093	O(1)	106.286	C(20)	110.446	Pro-R
36	H(36)	C(19)	1.1	O(6)	111.541	C(17)	105.987	Pro-S
37	H(37)	C(20)	1.092	O(7)	105.251	C(18)	110.973	Pro-S
38	H(38)	C(22)	1.103	O(8)	110.904	C(21)	107.377	Pro-S
39	H(39)	C(23)	1.102	O(5)	107.898	C(26)	109.156	Pro-R
40	H(42)	C(25)	1.092	O(9)	105.518	C(22)	107.82	Pro-R
41	H(43)	C(26)	1.09	O(10)	106.112	C(23)	109.996	Pro-S
42	H(44)	C(27)	1.099	O(4)	107.65	C(25)	109.845	Pro-R
43	H(45)	C(28)	1.107	O(11)	110.567	C(26)	107.668	Pro-R
44	H(40)	C(24)	1.095	O(3)	111.138	C(18)	107.639	Pro-R
45	H(41)	C(24)	1.093	O(3)	109.363	C(18)	109.991	Pro-S
46	H(46)	C(30)	1.094	O(2)	110.751	C(23)	110.216	Pro-R
47	H(47)	C(30)	1.097	O(2)	110.493	C(23)	107.116	Pro-S
48	H(48)	C(31)	1.096	O(13)	110.909	C(17)	109.643	Pro-R
49	H(49)	C(31)	1.093	O(13)	107.408	C(17)	109.281	Pro-S
50	H(50)	C(32)	1.097	O(14)	111.576	C(21)	108.783	Pro-S
51	H(51)	C(32)	1.09	O(14)	107.072	C(21)	108.509	Pro-R
52	H(52)	C(33)	1.098	O(15)	111.824	C(27)	108.799	Pro-S
53	H(53)	C(33)	1.1	O(15)	111.426	C(27)	107.598	Pro-R
54	H(55)	C(34)	1.093	O(16)	107.208	C(29)	107.569	Pro-R
55	H(56)	C(34)	1.095	O(16)	112.099	C(29)	109.507	Pro-S
56	H(54)	O(6)	0.962	C(19)	108.862	C(17)	-69.534	Dihedral
57	H(57)	O(7)	0.963	C(20)	108.274	C(18)	68.835	Dihedral
58	H(58)	O(8)	0.964	C(22)	106.752	C(21)	163.647	Dihedral
59	H(59)	O(9)	0.964	C(25)	108.292	C(22)	-64.816	Dihedral
60	H(60)	O(10)	0.965	C(26)	108.215	C(23)	56.995	Dihedral
61	H(61)	O(11)	0.964	C(28)	107.025	C(26)	46.26	Dihedral
62	H(62)	O(12)	0.971	C(29)	105.3	O(5)	-95.369	Dihedral
63	H(63)	O(13)	0.965	C(31)	105.605	C(17)	54.984	Dihedral
64	H(64)	O(14)	0.973	C(32)	110.397	C(21)	-49.203	Dihedral
65	H(65)	O(15)	0.961	C(33)	108.381	C(27)	-175.535	Dihedral
66	H(66)	O(16)	0.977	C(34)	106.977	C(29)	-78.61	Dihedral

Table S5: Optimized geometry parameters for the natural product Inulin from chicory.

S.no.	Atom	Bond Atom	Bond Length (Å)	Angle Atom	Angle (°)	2nd Angle Atom	2nd Angle (°)	2nd Angle Type
1	O(1)	-	-	-	-	-	-	-
2	C(12)	O(1)	1.408	-	-	-	-	-
3	C(13)	C(12)	1.559	O(1)	105.805	-	-	-

4	C(14)	C(13)	1.523	C(12)	102.352	O(1)	-26.029	Dihedral
5	C(15)	O(1)	1.446	C(12)	110.881	C(13)	3.13	Dihedral
6	O(2)	C(12)	1.436	O(1)	113.145	C(13)	110.751	Pro-R
7	C(16)	O(2)	1.402	C(12)	122.208	O(1)	23.884	Dihedral
8	O(3)	C(16)	1.418	O(2)	106.538	C(12)	-112.688	Dihedral
9	C(17)	C(16)	1.535	O(2)	108.687	O(3)	111.19	Pro-R
10	C(18)	C(17)	1.536	C(16)	110.457	O(2)	173.563	Dihedral
11	C(19)	C(18)	1.537	C(17)	110.584	C(16)	-50.24	Dihedral
12	C(20)	O(3)	1.425	C(16)	112.127	O(2)	179.313	Dihedral
13	O(4)	C(13)	1.416	C(12)	114.662	C(14)	112.609	Pro-R
14	O(5)	C(14)	1.421	C(13)	116.025	C(15)	109.129	Pro-S
15	O(6)	C(17)	1.412	C(16)	110.284	C(18)	108.86	Pro-S
16	O(7)	C(18)	1.414	C(17)	112.366	C(19)	112.406	Pro-R
17	O(8)	C(19)	1.413	C(18)	108.142	C(20)	111.168	Pro-S
18	C(21)	C(12)	1.527	O(1)	111.485	O(2)	103.47	Pro-R
19	C(22)	C(15)	1.525	O(1)	111.246	C(14)	114.491	Pro-S
20	C(23)	C(20)	1.532	O(3)	105.171	C(19)	113.136	Pro-S
21	O(9)	C(21)	1.426	C(12)	109.164	O(1)	179.381	Dihedral
22	O(10)	C(22)	1.416	C(15)	110.221	O(1)	171.028	Dihedral
23	O(11)	C(23)	1.43	C(20)	112.827	O(3)	-179.605	Dihedral
24	H(24)	C(13)	1.099	O(4)	110.188	C(12)	108.599	Pro-S
25	H(25)	C(14)	1.099	O(5)	110.803	C(13)	108.936	Pro-R
26	H(26)	C(15)	1.099	O(1)	109.474	C(14)	108.589	Pro-R
27	H(27)	C(16)	1.098	O(2)	110.848	O(3)	109.617	Pro-S
28	H(28)	C(17)	1.105	O(6)	111.565	C(16)	106.876	Pro-R
29	H(29)	C(18)	1.097	O(7)	104.953	C(17)	108.234	Pro-S
30	H(30)	C(19)	1.108	O(8)	111.311	C(18)	108.009	Pro-R
31	H(31)	C(20)	1.106	O(3)	110.265	C(19)	107.552	Pro-R
32	H(32)	C(21)	1.093	O(9)	107.283	C(12)	110.116	Pro-R
33	H(33)	C(21)	1.097	O(9)	112.197	C(12)	109.225	Pro-S
34	H(34)	C(22)	1.103	O(10)	112.095	C(15)	109.667	Pro-R
35	H(35)	C(22)	1.094	O(10)	107.325	C(15)	109.819	Pro-S
36	H(37)	C(23)	1.094	O(11)	106.111	C(20)	109.112	Pro-R
37	H(38)	C(23)	1.097	O(11)	111.58	C(20)	109.287	Pro-S
38	H(36)	O(4)	0.971	C(13)	105.917	C(12)	-59.939	Dihedral
39	H(39)	O(5)	0.967	C(14)	107.81	C(13)	72.197	Dihedral
40	H(40)	O(6)	0.975	C(17)	108.007	C(16)	60.78	Dihedral
41	H(41)	O(7)	0.967	C(18)	107.949	C(17)	65.133	Dihedral
42	H(42)	O(8)	0.972	C(19)	106.051	C(18)	-174.534	Dihedral
43	H(43)	O(9)	0.969	C(21)	105.234	C(12)	-66.028	Dihedral
44	H(44)	O(10)	0.97	C(22)	107.783	C(15)	44.423	Dihedral
45	H(45)	O(11)	0.967	C(23)	108.022	C(20)	-71.932	Dihedral

Table S6: Optimized geometry parameters for the natural product 1,3-Dicaffeoylquinic Acid.

S.no.	Atom	Bond Atom	Bond Length (Å)	Angle Atom	Angle (°)	2nd Angle Atom	2nd Angle (°)	2nd Angle Type
1	C(26)	-	-	-	-	-	-	-
2	C(28)	C(26)	1.408	-	-	-	-	-
3	C(29)	C(26)	1.401	C(28)	117.892	-	-	-
4	C(32)	C(28)	1.387	C(26)	122.089	C(29)	-0.058	Dihedral
5	C(33)	C(29)	1.388	C(26)	120.41	C(28)	0.063	Dihedral
6	C(36)	C(33)	1.395	C(29)	121.275	C(26)	-0.015	Dihedral
7	C(24)	C(26)	1.457	C(28)	118.549	C(29)	123.559	Pro-S
8	O(9)	C(32)	1.363	C(28)	123.742	C(36)	117.049	Pro-R
9	H(58)	O(9)	0.963	C(32)	108.944	C(28)	-0.714	Dihedral
10	O(11)	C(36)	1.359	C(32)	117.271	C(33)	123.604	Pro-S
11	H(60)	O(11)	0.963	C(36)	109.062	C(32)	-179.533	Dihedral
12	C(22)	C(24)	1.344	C(26)	127.967	C(28)	-179.489	Dihedral
13	C(20)	C(22)	1.473	C(24)	120.015	C(26)	179.88	Dihedral
14	O(1)	C(20)	1.364	C(22)	109.351	C(24)	-178.908	Dihedral
15	C(13)	O(1)	1.453	C(20)	121.916	C(22)	-177.131	Dihedral
16	C(14)	C(13)	1.556	O(1)	111.294	C(20)	61.532	Dihedral
17	C(16)	C(13)	1.538	O(1)	112.105	C(14)	111.211	Pro-R
18	C(15)	C(14)	1.545	C(13)	111.738	O(1)	-179.107	Dihedral
19	C(17)	C(15)	1.539	C(14)	112.414	C(13)	51.121	Dihedral
20	C(18)	C(16)	1.533	C(13)	110.839	O(1)	-178.494	Dihedral
21	O(2)	C(17)	1.452	C(15)	110.61	C(18)	106.699	Pro-S
22	O(3)	C(14)	1.41	C(13)	112.086	C(15)	112.554	Pro-R
23	O(4)	C(15)	1.415	C(14)	106.856	C(17)	112.551	Pro-S
24	C(19)	C(13)	1.545	O(1)	100.178	C(14)	110.925	Pro-S
25	O(7)	C(20)	1.21	O(1)	124.555	C(22)	126.09	Pro-R
26	C(21)	O(2)	1.355	C(17)	117.769	C(15)	88.408	Dihedral
27	C(23)	C(21)	1.468	O(2)	110.719	C(17)	177.147	Dihedral
28	O(8)	C(21)	1.217	O(2)	123.325	C(23)	125.953	Pro-R
29	C(25)	C(23)	1.344	C(21)	120.115	O(2)	-179.462	Dihedral
30	C(27)	C(25)	1.456	C(23)	127.944	C(21)	-179.873	Dihedral
31	C(30)	C(27)	1.408	C(25)	118.537	C(23)	-179.947	Dihedral

32	C(31)	C(27)	1.402	C(25)	123.519	C(30)	117.944	Pro-R
33	C(34)	C(30)	1.387	C(27)	122.056	C(25)	-179.912	Dihedral
34	C(35)	C(31)	1.388	C(27)	120.389	C(25)	179.929	Dihedral
35	C(37)	C(35)	1.395	C(31)	121.258	C(27)	-0.019	Dihedral
36	O(10)	C(34)	1.363	C(30)	123.787	C(37)	117.023	Pro-R
37	H(59)	O(10)	0.963	C(34)	109.008	C(30)	-0.542	Dihedral
38	O(12)	C(37)	1.359	C(34)	117.258	C(35)	123.579	Pro-S
39	H(61)	O(12)	0.963	C(37)	109.071	C(34)	-179.72	Dihedral
40	O(5)	C(19)	1.344	C(13)	112.227	O(1)	67.516	Dihedral
41	O(6)	C(19)	1.208	O(5)	122.118	C(13)	125.655	Pro-S
42	H(52)	C(28)	1.087	C(26)	119.078	C(32)	118.833	Pro-S
43	H(53)	C(29)	1.082	C(26)	120.418	C(33)	119.172	Pro-R
44	H(54)	C(30)	1.087	C(27)	119.089	C(34)	118.855	Pro-S
45	H(55)	C(31)	1.082	C(27)	120.445	C(35)	119.165	Pro-R
46	H(56)	C(33)	1.086	C(29)	119.871	C(36)	118.854	Pro-R
47	H(57)	C(35)	1.086	C(31)	119.887	C(37)	118.855	Pro-R
48	H(48)	C(22)	1.082	C(20)	116.564	C(24)	123.421	Pro-R
49	H(49)	C(23)	1.082	C(21)	116.45	C(25)	123.434	Pro-R
50	H(50)	C(24)	1.088	C(22)	115.99	C(26)	116.043	Pro-R
51	H(51)	C(25)	1.088	C(23)	116.011	C(27)	116.046	Pro-S
52	H(38)	C(14)	1.089	O(3)	105.877	C(13)	106.986	Pro-S
53	H(39)	C(15)	1.101	O(4)	110.072	C(14)	108.638	Pro-R
54	H(40)	C(16)	1.089	C(13)	107.791	C(18)	109.58	Pro-R
55	H(41)	C(16)	1.091	C(13)	109.241	C(18)	110.97	Pro-S
56	H(42)	C(17)	1.092	O(2)	107.516	C(15)	109.018	Pro-R
57	H(43)	C(18)	1.096	C(16)	111.008	C(17)	108.365	Pro-S
58	H(44)	C(18)	1.092	C(16)	109.92	C(17)	109.063	Pro-R
59	H(45)	O(3)	0.965	C(14)	107.085	C(13)	66.662	Dihedral
60	H(46)	O(4)	0.966	C(15)	108.324	C(14)	-153.577	Dihedral
61	H(47)	O(5)	0.97	C(19)	106.698	O(6)	2.344	Dihedral

Table S7: Optimized geometry parameters for the reference compound 6-(ethylamino)pyridine-3-carbonitrile.

S.no.	Atom	Bond Atom	Bond Length (Å)	Angle Atom	Angle (°)	2nd Angle Atom	2nd Angle (°)	2nd Angle Type
1	N(2)	-	-	-	-	-	-	-
2	C(5)	N(2)	1.35	-	-	-	-	-
3	C(7)	C(5)	1.415	N(2)	122.051	-	-	-
4	C(8)	C(7)	1.378	C(5)	118.507	N(2)	0	Dihedral
5	C(9)	C(8)	1.406	C(7)	119.764	C(5)	-0.005	Dihedral
6	C(10)	N(2)	1.323	C(5)	118.497	C(7)	0.005	Dihedral
7	N(1)	C(5)	1.358	N(2)	115.324	C(7)	122.626	Pro-R
8	C(11)	C(9)	1.423	C(8)	121.799	C(10)	120.978	Pro-S
9	C(4)	N(1)	1.453	C(5)	125.915	N(2)	-179.985	Dihedral
10	C(6)	C(4)	1.525	N(1)	110.436	C(5)	179.984	Dihedral
11	N(3)	C(11)	1.157	C(9)	179.861	C(8)	178.348	Dihedral
12	H(18)	C(7)	1.081	C(5)	120.989	C(8)	120.505	Pro-S
13	H(19)	C(8)	1.083	C(7)	120.39	C(9)	119.846	Pro-R
14	H(20)	C(10)	1.086	N(2)	116.366	C(9)	119.675	Pro-R
15	H(12)	C(4)	1.098	N(1)	109.984	C(6)	109.71	Pro-S
16	H(13)	C(4)	1.098	N(1)	109.985	C(6)	109.705	Pro-R
17	H(14)	N(1)	1.009	C(4)	119.374	C(5)	114.712	Pro-R
18	H(15)	C(6)	1.093	C(4)	110.964	N(1)	-60.322	Dihedral
19	H(16)	C(6)	1.093	C(4)	110.957	H(15)	108.455	Pro-S
20	H(17)	C(6)	1.091	C(4)	110.361	H(15)	108	Pro-R

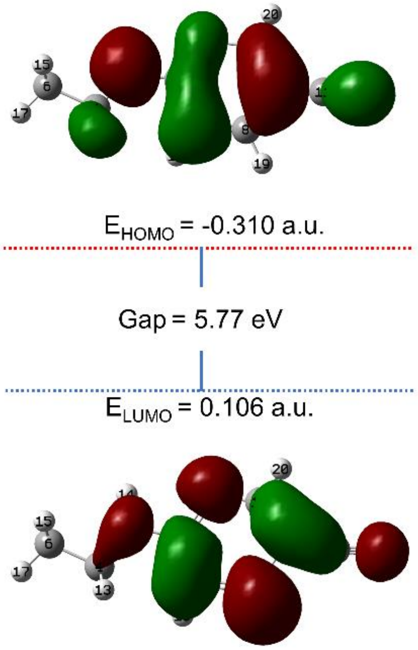

Figure S1. Molecular orbitals LUMO and HOMO of the optimized reference compound 6-(ethylamino)pyridine-3-carbonitrile calculated using theoretical model B3LYP/6-31G**.

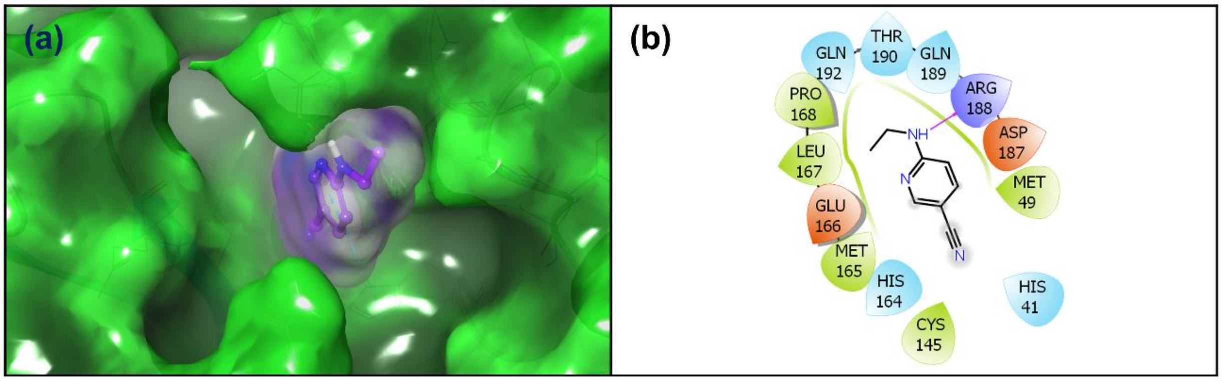

Figure S2. 2D interaction maps for the docked poses of SARS-CoV-2 M^{pro} with reference compound 6-(ethylamino)pyridine-3-carbonitrile. In 3D interaction poses, docked ligand surface was generated based on partial charge while protein surface was rendered based on secondary structure. While in 2D maps, hydrogen bond formation (pink arrows), hydrophobic (green), polar (blue), red (negative), and violet (positive) interaction are logged for docked complexes of SARS-CoV-2 M^{pro} with reference compound.

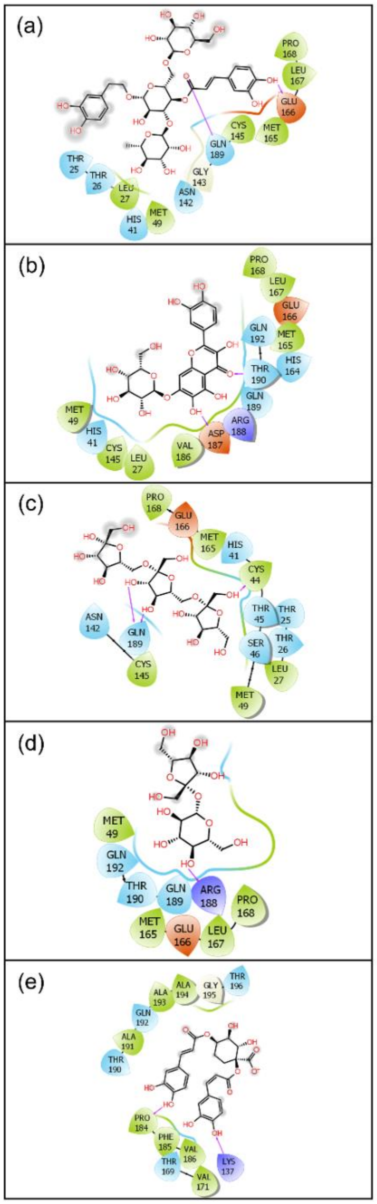

Figure S3. 2D interaction maps for the docked poses of SARS-CoV-2 Mpro with potent compounds, i.e. (a-b) Levan N, (c-d) Echinacoside, (e-f) Quercetagetin 7-Glucoside, (g-h) Inulin From Chicory, and (i-j) 1,3-Dicaffeoylquinic Acid after MD simulation. In 2D maps, hydrogen bond formation (pink arrows), hydrophobic (green), polar (blue), red (negative), violet (positive), glycine (grey) and π-π stacking (green line) interaction are logged for docked complexes of SARS-CoV-2 M^{pro} with selected natural compounds.

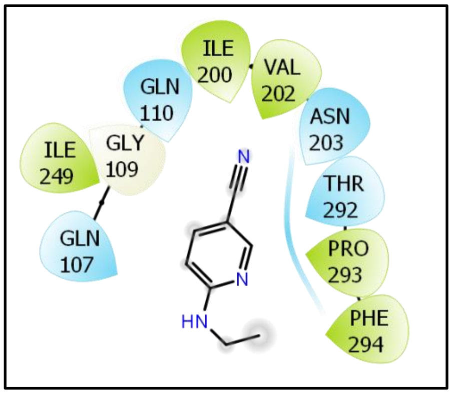

Figure S4. 2D interaction maps for the docked poses of SARS-CoV-2 M^{pro} with reference compound 6-(ethylamino)pyridine-3-carbonitrile after MD simulation. In 3D interaction poses, docked ligand surface was generated based on partial charge while protein surface was rendered based on secondary structure. While in 2D maps, hydrogen bond formation (pink arrows), hydrophobic (green), polar (blue), red (negative), and violet (positive) interaction are logged for docked complexes of SARS-CoV-2 M^{pro} with reference compound.

Table S8. List of interactions and residues at the active pocket of viral protease interacting with the potential natural compounds logged from respective docked complexes after 100 ns simulation.

S. no.	Drug	H-Bond	π-π Stacking	Hydrophobic	Polar	Negative	Positive	Glycine
1.	Echinacoside	Glu^{166}, Gln^{189}	-	Leu^{27}, Met^{49}, Cys^{145}, Met^{165}, Pro^{168}, Leu^{167}	Thr^{25}, $Thr^{26,}$ His^{41}, Asn^{142}, Gln^{189}	Glu^{47}, Glu^{166}	-	Gly^{143}
2.	Quercetagetin 7-Glucoside	Asp^{187}, Thr^{190}	-	Leu^{27}, Met^{49}, Cys^{145} Met^{165}, Leu^{167}, Pro^{168}, Val^{186}	His^{41}, His^{164}, Gln^{189}, Gln^{192}, Thr^{190}	Glu^{166}, Asp^{187}	Arg^{188}	-
3.	Levan N	Cys^{44}, Gln^{189}	-	Leu^{27}, Cys^{44}, Met^{49}, Cys^{145}, Met^{165}, Pro^{168}	Thr^{25}, Thr^{26}, His^{41}, Thr^{45}, Ser^{46}, Asn^{142}, Gln^{189}	Glu^{166}	-	-
4.	Inulin from chicory	Arg^{188}	-	Met^{49}, Met^{165}, Leu^{167}, Pro^{168}	Gln^{189}, Thr^{190}, Gln^{192}	Glu^{166}	Arg^{188}	-
5.	1,3-Dicaffeoylquinic Acid	Lys^{137}, Pro^{184}	-	Val^{171}, Pro^{184}, Phe^{185}, Val^{186}, Ala^{191}, Ala^{193}, Ala^{194}	Thr^{169}, Thr^{190}, Gln^{192}, Thr^{196}	-	Lys^{137}	Gly^{195}
6.	6-(ethylamino)pyridine-3-carbonitrile	-	-	Ile^{200}, Val^{202}, Ile^{249}, Pro^{293}, Phe^{294}	Gln^{107}, Gln^{110}, Asn^{203}, Thr^{292}	-	-	Gly^{109}

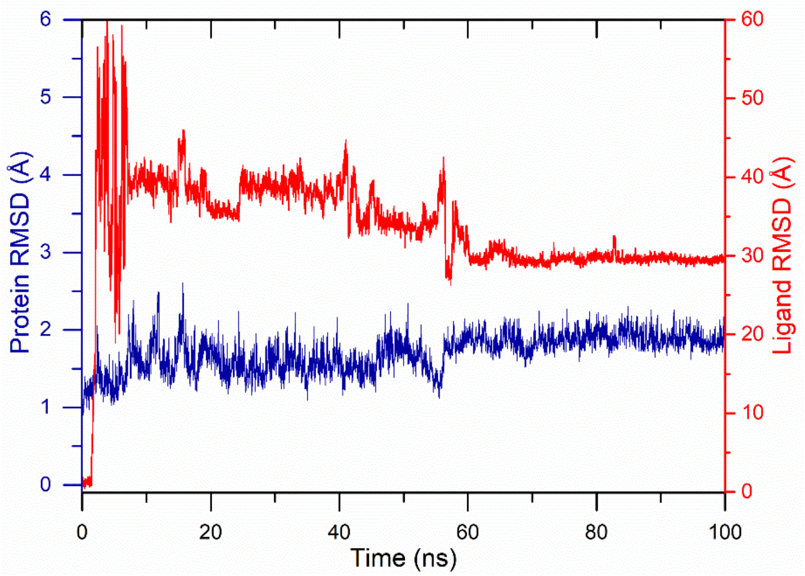

Figure S5. RMSD values plotted for alpha carbon atoms (blue curves) of SARS-CoV-2 M^{pro} and reference ligand 6-(ethylamino)pyridine-3-carbonitrile (red curves) for the respective docked complexes extracted from 100 ns MD simulation interval.

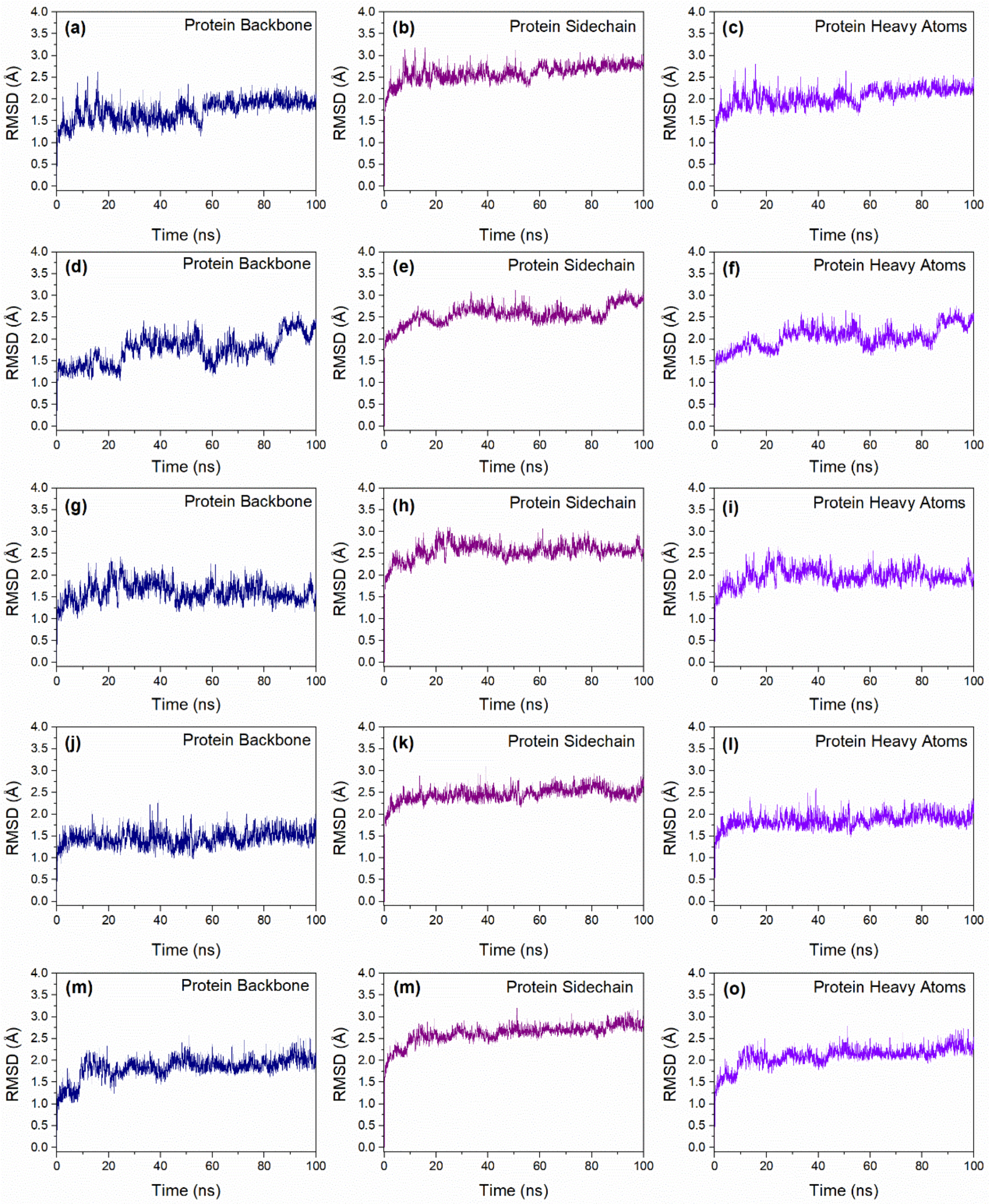

Figure S6. RMSD values plotted for Protein Backbone, Sidechains, and heavy Atoms in docked state during with (a-c) Echinacoside, (d-f) Quercetagetin 7-Glucoside, (g-i) Levan N, (j-l) Inulin from chicory, and (m-o) 1,3-Dicaffeoylquinic Acid, extracted from 100 ns MD simulation interval.

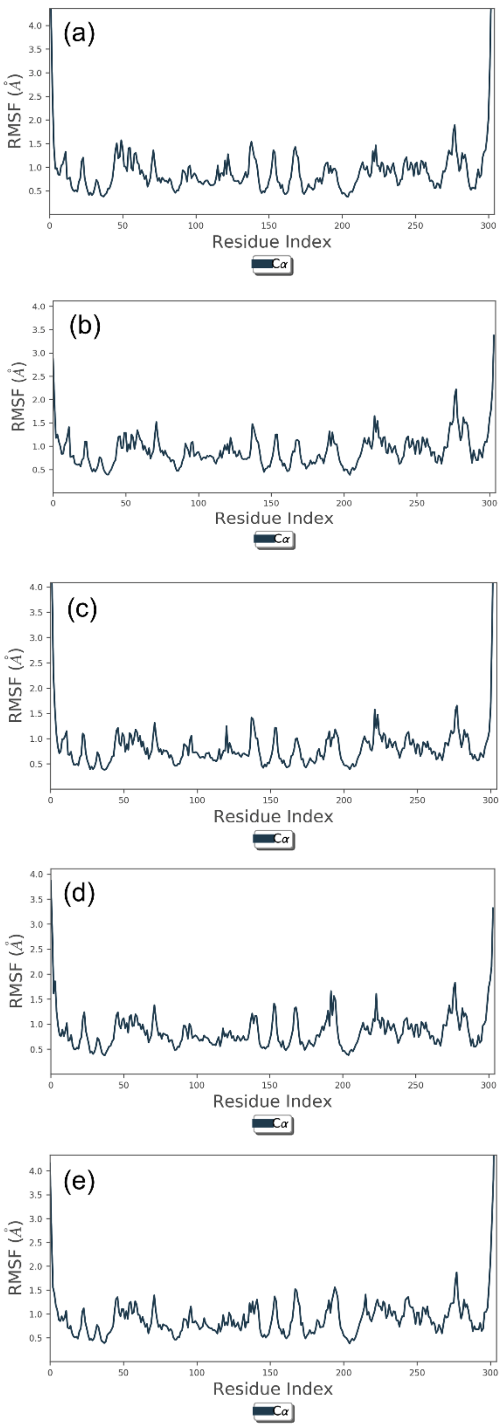

Figure S7. RMSF values plotted for alpha carbon atoms of SARS-CoV-2 M^{pro} docked with natural products i.e. (a) Echinacoside, (b) Quercetagetin 7-Glucoside, (c) Levan N, (d) Inulin from chicory, and (e) 1,3-Dicaffeoylquinic Acid, extracted from 100 ns MD simulation interval.

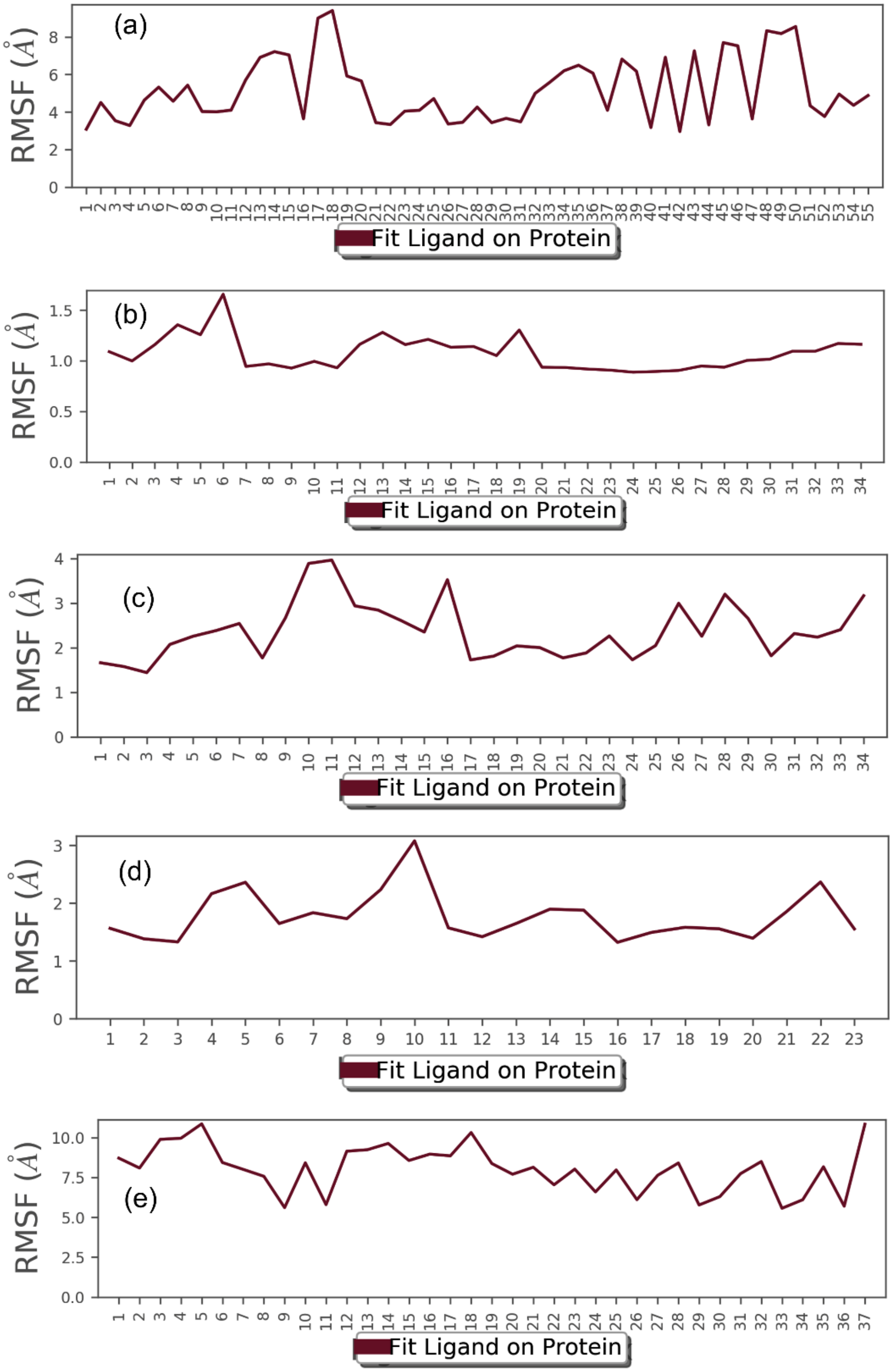

Figure S8. RMSF values plotted for the natural products i.e. (a) Echinacoside, (b) Quercetagetin 7-Glucoside, (c) Levan N, (d) Inulin from chicory, and (e) 1,3-Dicaffeoylquinic Acid fit with SARS-CoV-2 M^{pro}, extracted from 100 ns MD simulation interval.

Table. S9 Averaged binding free energies (kcal/mol) and their components calculated using MM/GBSA method for all the selected natural products docked with SARS-CoV-2 M^{pro}.

**Components**	**Energy (Kcal/mol)**				
	**Echinacoside**	**Quercetagetin 7-Glucoside**	**Levan N**	**Inulin From Chicory**	**1,3-Dicaffeoylquinic Acid**
MMGBSA ΔG Bind	-43.77±13.84	-65.82±2.74	-44.44±5.80	-50.53±9.75	-42.57±8.99
MMGBSA ΔG Bind Coulomb	-28.65±11.64	-34.62±4.30	-30.80±5.93	-36.61±6.16	8.72±10.33
MMGBSA ΔG Bind Covalent	3.36±2.36	2.84±1.12	3.18±2.04	2.80±1.48	3.50±5.42
MMGBSA ΔG Bind Hbond	-3.02±1.05	-3.99±0.20	-2.14±0.93	-3.85±0.65	-1.96±0.82
MMGBSA ΔG Bind Lipo	-9.63±3.140	-11.07±2.43	-9.22±1.73	-8.53±1.43	-12.41±3.15
MMGBSA ΔG Bind Packing	-1.23±1.06	-2.43±0.50	0±0	0±0	-1.02±0.70
MMGBSA ΔG Bind Solv GB	30.38±10.48	29.05±2.02	29.47±4.31	23.49±4.88	-5.04±9.86
MMGBSA ΔG Bind vdW	-34.9±8.09	-45.64±2.92	-34.94±3.50	-27.82±4.52	-34.36±6.62
Ligand Strain Energy	14.49±5.19	4.41±1.14	3.47±2.85	4.17±3.40	6.61±5.82
